# Promising Cellular Immunotherapy for Colorectal Cancer Using Classical Dendritic Cells and Natural Killer T Cells

**DOI:** 10.3390/cells14030166

**Published:** 2025-01-22

**Authors:** Mahmoud Singer, Jennifer Valerin, Zhuoli Zhang, Zigeng Zhang, Farshid Dayyani, Vahid Yaghmai, April Choi, David Imagawa, Nadine Abi-Jaoudeh

**Affiliations:** 1 Department of Radiological Sciences, School of Medicine, University of California, Irvine, CA 92617, USA; 2 Department of Medicine, Chao Family Comprehensive Cancer Center, University of California, Irvine, CA 92697, USAaprilc@hs.uci.edu (A.C.); 3 Department of Surgery, University of California Irvine, Orange, CA 92697, USA

**Keywords:** colorectal cancer, CRC, classical dendritic cells, NKT cells, NK cells, tumor microenvironment, TME modulation, cellular immunotherapy

## Abstract

Colorectal cancer (CRC) remains one of the leading causes of cancer-related morbidity and mortality around the world. Despite advances in surgery, chemotherapy, and targeted therapies, the prognosis for patients with metastatic or advanced CRC remains poor. Immunotherapies comprising immune checkpoint inhibitors showed disappointing responses in metastatic CRC (mCRC). However, cellular immunotherapy, specifically using classical dendritic cells (cDCs), may hold unique promise in immune recognition for CRC antigens. cDCs are substantial players in immune recognition and are instrumental in orchestrating innate and adaptive immune responses by processing and presenting tumor antigens to effector cells. Natural killer T (NKT) cells are insufficiently studied but unique effector cells because of their ability to bridge innate and adaptive immune reactions and the crosstalk with dendritic cells in cancer. This review explores the therapeutic potential of using both cDCs and NKT cells as a synergistic therapy in CRC, focusing on their biological roles, strategies for harnessing their capabilities, clinical applications, and the challenges within the tumor microenvironment. Both cDCs and NKT cells can be used as a new effective approach for cell-based therapies in cancers to provide a new hope for CRC patients that are challenging to treat.

## 1. Introduction

Colorectal cancer (CRC) is the third most commonly diagnosed cancer globally, with an estimated 1.93 million new cases and is the second most common cause of death (0.94 million annually) according to the 2020 worldwide statistics [1,2,3]. Despite advances in early detection methods and improvements in surgical techniques, chemotherapy, and targeted therapies, metastatic CRC (mCRC) remains challenging to treat, with failure to achieve long-term remission, especially in patients with advanced disease. Hence, mCRC remains a fatal disease with a 5-year survival rate of 14% [4,5,6]. Novel therapeutic strategies are needed. Immunotherapy has revolutionized cancer treatment, with checkpoint inhibitors and adoptive T cell therapies showing considerable promise in various malignancies [7,8]. However, their application in CRC has been fraught with challenges, including a complex tumor microenvironment and a low tumor mutational burden, especially in microsatellite-stable (MSS) patients who constitute 96% of mCRC cases [9,10].

We searched for up-to-date cellular immunotherapies for CRC and found that the current cellular immunotherapies are not effective as hoped. We searched the literature for studies on the main antigen-presenting cells that normally exist in cellular and humoral immunity that is affected by cancers: classical dendritic cells. Recently, natural killer T cells were found to have a merge of the biological benefits of both T cells and NK cells. This review aims to provide an overview of the status of immunotherapy for CRC, the challenges that hinder its effectiveness, and the potential strategies to overcome these barriers that depend on dendritic cells and NKT cells, with a focus on their biological characteristics, mechanisms of anti-tumor activity, therapeutic potential, and challenges in clinical application.

## 2. Why Is Colorectal Cancer Difficult to Treat?

**Figure 1:**
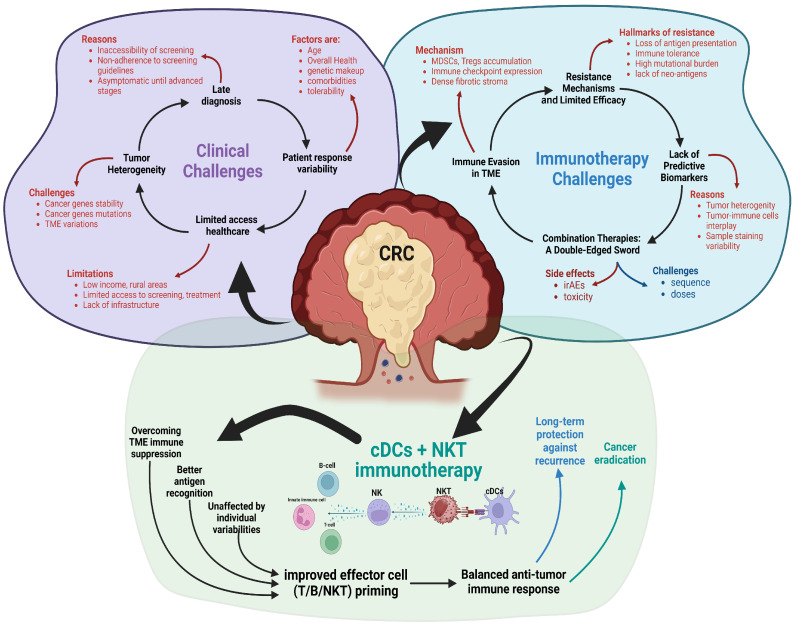
Representative scheme of the clinical and immunotherapeutic challenges for treating CRC.

The difficulty of managing CRC stems from several factors, including its complex biology, diagnosis at a late stage, treatment resistance, and patient-related issues (as shown in Figure 1).

### 2.1. CRC Tumor Heterogeneity and Complex Biology Related to Resistance

Colorectal cancer is not a single disease but rather a collection of disorders that share similar features but are biologically distinct. Tumors can arise from a variety of mutations in genes that control cell growth, apoptosis (programmed cell death), and DNA repair [11,12]. The most well-known genetic pathway involves mutations in the APC gene, which lead to the formation of polyps that can eventually become cancerous. However, CRC can also be driven by mutations in the KRAS, TP53, and BRAF genes, among others [13,14]. These mutations affect different signaling pathways, leading to various subtypes of the cancer with distinct behaviors and prognoses. This genetic heterogeneity complicates treatment, as therapies targeting specific genetic alterations (such as KRAS or BRAF inhibitors) may not be effective for all patients [15]. To further complicate things, there are mutation subtypes. For example, KRAS has numerous subtypes with varying tumoral behaviors and therapeutic responses [16]. Furthermore, tumors often acquire additional mutations during progression, making them even more difficult to treat.

The molecular classification of CRC includes microsatellite-stable (MSS) tumors and tumors with microsatellite instability (MSI), with the latter being further divided into high (MSI-H) and low (MSI-L) levels of instability [17,18]. Tumors with high microsatellite instability exhibit a higher mutational burden, which is positively correlated with the production of neo-antigens and pro-inflammatory tumor microenvironments [19]. This is because of the absence of DNA repair genes (also called mismatch repair (MMR) genes) in tumor cells and defects in the replication repair process, which can lead to the formation of neo-antigens that the immune system recognizes as foreign [20]. These tumors tend to respond better to immune checkpoint inhibitors due to their increased immunogenicity [21,22]. The fact that MSS tumors have a high mutation burden makes them less visible to the immune system and they can evade immune detection more effectively, resulting in increased resistance to immune checkpoint inhibitors. Unlike those with microsatellite instability (MSI), tumors that are highly responsive to immunotherapy have numerous mutations, making it easy for the immune system to detect them [19]. Microsatellite-stable tumors, in contrast, exhibit a relatively lower mutational burden and less immunogenicity, which makes them more resistant to immune checkpoint inhibitors. The challenge here is that these tumors often have mechanisms that suppress immune activation, making them harder to treat with current immunotherapies [23,24]. Tumor heterogeneity extends beyond genetic mutations; it also involves the composition of the immune cells, stromal cells, and cytokines that shape the environment, called the tumor microenvironment (TME). The heterogeneity of the TME complicates the identification of biomarkers for patient stratification and reduces the overall success rate of therapies [25]. An example of tumors’ inherent heterogeneity is the cancer cell’s evolution over time, where they acquire mutations for their survival and develop mechanisms that prevent drug uptake or repair the damage caused by the therapy [26]. Immunotherapy, a newer treatment option, has shown promise for certain subsets of colorectal cancer, particularly those with high microsatellite instability (MSI-H). However, only a minority of patients exhibit this characteristic, leaving many others without an effective treatment option [27,28]. Targeted therapies have improved treatments for some patients, particularly those with specific genetic mutations, but not all patients respond, and resistance can develop over time [29].

### 2.2. Late-Stage Diagnosis

The diagnosis at a later stage is another challenge with CRC as patients remain asymptomatic until advanced stages of the disease. This is particularly true for cancers on the right side of the colon. Screening programs, such as colonoscopies and fecal occult blood tests, have helped in detecting CRC early, but these methods are not universally accessible, and adherence to screening guidelines remains a challenge. As a result, many patients are diagnosed too late for curative treatment. As a result, the diagnosis occurs at an advanced stage, limiting treatment options and decreasing survival [30,31,32,33].

### 2.3. Variability in Patient Response

The effectiveness of CRC treatment also varies greatly depending on individual patient factors. Age, overall health, comorbidities, and genetic makeup all influence patient responses [34,35] and tolerance. Furthermore, genetic differences between individuals can result in varying drug metabolism rates, making it difficult to predict the best course of action for each patient [36,37].

### 2.4. Limited Access to Healthcare

Limited access to healthcare is one of the challenges for treating CRC patients. In many low-income or rural areas, patients may have limited access to screening, timely diagnosis, or advanced treatments, such as surgery or personalized therapies. The lack of healthcare infrastructure exacerbates the challenge, leading to delayed diagnoses and poorer outcomes [38,39].

## 3. Key Challenges in Immunotherapy for CRC

### 3.1. Immune Evasion and the Tumor Microenvironment

CRC tumors, particularly MSS tumors, create an immunosuppressive microenvironment that prevents the immune system from effectively attacking cancer cells [40]. Several factors contribute to this immune escape, such as immune checkpoint molecules which bind to PD-1 on T cells, leading to T cell exhaustion and inhibition of immune responses. The presence of these molecules within the TME can blunt the effectiveness of immune checkpoint inhibitors [41]. CRC tumors often harbor a high number of regulatory T cells (Tregs), which actively suppress the function of cytotoxic T cells and limit the ability of the immune system to target tumor cells. The accumulation of Tregs within the TME is a major barrier to the success of immunotherapy [42,43]. Myeloid-derived suppressor cells (MDSCs) are another key component of the immunosuppressive TME. These cells inhibit the activation of T cells and promote tumor progression by secreting immunosuppressive cytokines [44]. The known signaling pathways through which MDSCs suppress T cell function are one of the following eight possibilities or a combination of them: (1) consumption of T cell nutrients such as arginase-1 and nitric oxide synthase—arginase-1 deficiency depletes L-arginine which is required for T cell proliferation [45,46,47]; (2) generation of reactive oxygen species (ROS), which impair T cell function and induces naïve CD4 T cells to differentiate into Tregs [48]; (3) secretion of immunosuppressive cytokines by MDSCs such as TGF-b, IL-10, vascular endothelial growth factor (VEGF), and prostaglandin E2 (PGE2) [49]; (4) expression of immune checkpoint molecules such as programmed Death Ligand 1 (PD-L1) and CTLA-4 [50]; (5) induction of Tregs via TGF-b and IL-10 secretion [43,51]; (6) excessive conversion of ATP to adenosine by MDSCs via the CD39/CD73 pathway, which leads to reduced TCR signaling and suppressed proliferation [52,53]; (7) reduction in the secretion of matrix metalloproteinases (MMPs), preventing T cell infiltration and downregulating T cell chemotaxis [54]; and (8) induction of T cell apoptosis via the binding of FasL expressed on MDSCs to the TNF-Related Apoptosis-Inducing Ligand (TRIAL) death receptors on T cells [55,56]. The presence of dense fibrotic stroma in CRC can act as a physical barrier to immune cell infiltration. This restricts the access of immune cells, including T cells and dendritic cells, to the tumor site, thus limiting the effectiveness of immunotherapies [57].

### 3.2. Immunotherapy Resistance Mechanisms and Limited Efficacy in MSS CRC

Resistance can develop over time, even among patients who initially respond to immunotherapy. The mechanisms of resistance include downregulating the expression of major histocompatibility complex (MHC) molecules and alterations in antigen presentation. Loss of antigen presentation can lead to immune escape, even in the presence of immune checkpoint inhibitors [58]. The molecular basis of impaired antigen presentation in cancer is a multifaceted process. It includes defects in MHC I expression due to mutations in the genes encoding the MHC I heavy chains, TAP, tapasin, ß2M, ERAP1, and subunits of the immunoproteasome, ultimately leading to a decrease in MHC I surface levels and an increase in PD-L1 levels [59]. Another mechanism is through epigenetic silencing due to hypermethylation of the promoters of the regulatory elements of MHC I, TAP, tapasin, and IFNR pathway components [60,61,62,63]. Another possible mechanism of resistance is the upregulation of alternative immune checkpoint proteins, such as TIM-3, LAG-3, and VISTA, as well as PD-1/PD-L1, which may become upregulated in response to immune checkpoint blockade. These alternative checkpoints can limit the effectiveness of current therapies and contribute to acquired resistance [64,65].

Some CRC tumors may exploit immune tolerance mechanisms, where the immune system is “re-educated” to accept the tumor as a normal part of the body. This can occur through the induction of immune regulatory networks that suppress the activation of anti-tumor immunity [66,67]. The majority of CRCs are MSS, and these tumors tend to be less responsive to immunotherapy compared to MSI-H tumors. While there is some evidence that combining immunotherapy with other treatments may enhance responses in MSS tumors, they remain a difficult challenge. This is due to their relatively low mutational burden, lack of neo-antigens, and immunosuppressive TME. Innovative strategies are needed to overcome the resistance mechanisms specific to MSS CRC [68,69].

### 3.3. Lack of Predictive Biomarkers

A major challenge is the lack of reliable predictive biomarkers that can accurately identify patients who will respond to treatment. While MSI-H and mismatch repair-deficient (dMMR) statuses are strong indicators of a response to immune checkpoint inhibitors, the vast majority of CRC cases are MSS and mismatch repair-proficient (pMMR), and these patients are less likely to benefit from immunotherapy [70,71]. Several biomarkers are being investigated for their ability to predict response to immunotherapy in CRC. The Tumor Mutational Burden (TMB) is one of the biomarkers used, which is associated with an increased likelihood of response to immune checkpoint inhibitors as tumors with a high mutational load are more likely to present novel antigens to the immune system [72]. However, the TMB is not universally predictive across all cancer types, and its utility in CRC is still under investigation. While PD-L1 expression is often used as a biomarker for response to PD-1/PD-L1 inhibitors, its predictive value in CRC is inconsistent because of the tumor heterogeneity, complex interplay between tumor cells and immune cells expressing PD-L1, and technical variability in sample staining [71,73]. The relationship between PD-L1 expression and clinical outcomes in CRC is complex, and it is not always a reliable marker for identifying patients who will benefit from immunotherapy [74]. The composition of immune cells within the TME may provide insights into the likelihood of a response to immunotherapy. High levels of cytotoxic T cells and a favorable Th1/Th2 ratio correlate with better responses to immunotherapy. However, quantifying immune cell infiltration and understanding the complex interactions between immune cells within the TME remain a challenge in order to create a prognostic marker for CRC tumors [75].

### 3.4. Combination Therapies: A Double-Edged Sword

The increase interest in combination therapies to enhance treatment efficacy, such as adding immune checkpoint inhibitors to chemotherapy, targeted therapy, or radiation, holds promise. However, combining therapies also introduces new challenges in reducing toxicity and side effects and determining the optimal sequencing and dosing [76,77]. Combination therapies can lead to increased toxicity, particularly when combining immune checkpoint inhibitors with cytotoxic agents. Immune-related adverse events (irAEs), such as colitis, pneumonitis, and hepatitis, are a concern with immune checkpoint blockade, and these side effects may be exacerbated when combined with other therapies [78,79]. Determining the most effective combination regimen, sequencing, and dosing remains an ongoing challenge. While preclinical studies suggest that certain combinations may be more effective than others, there is still a lack of clinical data to definitively identify the best approach [80].

### 3.5. Updates to Immunotherapy for CRC

Immunotherapy, which aims to enhance the body’s immune system to recognize and eliminate cancer cells, has revolutionized cancer treatment over the past decade [81]. In CRC, immunotherapy has primarily focused on two main approaches:

1. Immune Checkpoint Inhibitors (ICIs), i.e., monoclonal antibodies, block immune checkpoint proteins, such as PD-1/PD-L1 and CTLA-4, allowing effector cells to become activated and attack tumor cells [21]. Among these, immune checkpoint inhibitors, pembrolizumab (anti-PD-1) and nivolumab (another anti-PD-1) have shown efficacy in high microsatellite instability (MSI-H) CRC, a subset of CRC with a high mutational burden that is particularly responsive to immunotherapy. However, only 4% of metastatic CRC cases are MSI-H and could therefore benefit from these treatments [71,82,83]. The interaction between PD-1 and its ligands primarily impacts CD8+ T cells, the key players in anti-tumor immunity, resulting in reduced cytotoxic activity. Normally, PD-1’s main job is to send signals that quiet down T cells during an immune response. It achieves this by blocking the activity of casein kinase 2 (CK2), ensuring that the immune system does not become overly aggressive. This inhibition blocks the phosphorylation of PTEN’s regulatory domain, halting phosphoinositide 3-kinase (PI3K) activity, inhibiting cyclin-dependent kinase (CDK), and modulating T cell receptor expression [84]. Tumor necrosis factor alpha (TNF-α) and interleukin-17 (IL-17) are mainly affected by anti-PD-1/anti-PD-L1 therapies because they contribute to the expression of PD-L1 in both tumor and immune cells [85].
  Phase II [86] and phase III [87] clinical trials using the anti-CTLA-4 antibody “ipilimumab” alone or with combination with the anti-PD-L1 antibody “nivolumab” showed that at least 30% of dMMR/MSI-H mCRC patients do not respond to these ICI treatment, and more than 20% of patients are refractory to this therapy. This refractory response was explained by the mutations found in the HLA-A, -B, and -C loci. In another phase III clinical trial, when treating advanced and metastatic CRC patients with the IgG-4-based anti-PD-1 monoclonal antibody “pembrolizumab”, 22% of the ICI-treated group suffered from the side effects and some patients died during the study [88].
  Cancer vaccines and oncolytic viruses aim to stimulate the immune system to target cancer-specific antigens, either through vaccine-induced immune responses or with direct infection of cancer cells using modified viruses [89]. Theoretically, the development of an anti-cancer vaccine hinges on leveraging overexpressed proteins, cancer/testis antigens, oncoviral antigens, shared cancer neo-antigens, or cancer-specific antigens to enhance the immune response against colorectal cancer (CRC) tumors [90]. Previous preclinical and clinical trials of cancer vaccines have neglected key immunological factors used for anti-viral vaccines, including the balance of immune responses, the nature of innate and adaptive immunity, the generation of long-lasting memory cells, and the production of chemokines, which are all crucial factors for evaluating vaccine success [91,92,93,94].
2. Cellular immunotherapy uses dendritic cells (DCs), which specialize in capturing and processing tumor-associated antigens (TAAs), presenting these antigens to effector cells, and promoting the activation of an adaptive immune response against tumors [95]. With advances in DC-based vaccine development, the therapeutic potential of cDCs in CRC has become a major area of investigation. One of the important unstudied effector cells is natural killer T (NKT) cells, an important subset of lymphocytes due to their ability to recognize and respond to both tumor- and pathogen-derived antigens. Unlike conventional T cells, which rely on specific peptide antigens presented by MHC molecules, NKT cells recognize lipid antigens presented by CD1d molecules, making them a unique and versatile tool for immunotherapy [96,97]. NKT cells could be a novel approach for personalized cellular therapy; however, this therapy generally takes time to produce and administer to patients, particularly those who have rapidly progressing diseases [98,99].

## 4. Classical Dendritic Cells in Immunotherapy for CRC

### 4.1. The Immune System and Dendritic Cells

**Figure 2:**
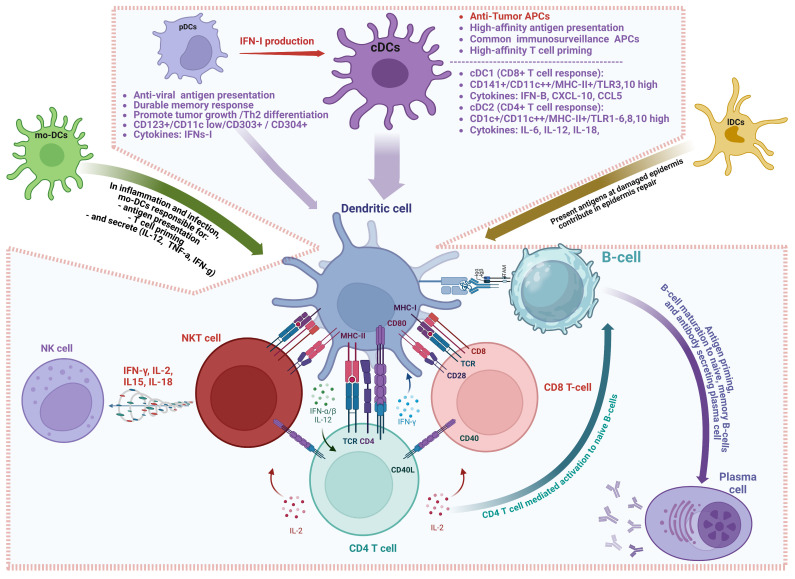
Dendritic cell subsets and their impact on antigen presentation.

Dendritic cells are pivotal players in the initiation of immune responses. They are classified into four main categories: plasmacytoid dendritic cells (pDCs), monocytic-derived dendritic cells (mo-DCs), Langerhans dendritic cells (lDCs), and classical dendritic cells (cDCs) [100]. cDCs are highly efficient in antigen presentation through both the major histocompatibility complex (MHC) class I and class II pathways, enabling them to stimulate cytotoxic CD8+ T cells and helper CD4+ T cells, respectively [101]. cDCs specialize in interacting with multiple immune components, including T cells, natural killer (NK) cells, macrophages, and B cells. Their ability to prime T cells and recruit other immune cells to the tumor site is crucial for the success of anti-cancer immunity [95]. The dynamic interaction between cDCs and other immune cells within the TME can significantly impact the effectiveness of immunotherapies [102] (Figure 2).

### 4.2. Classical Dendritic Cells and Tumor Antigen Presentation

The core function of cDCs in anti-tumor immunity lies in their capacity to present tumor-associated antigens (TAAs) to naïve T cells. Tumor cells often express abnormal proteins or mutated antigens that are not present on normal cells, allowing cDCs to capture and process these tumor-specific antigens. Once processed, these antigens are presented on the surface of cDCs via MHC class I (for CD8+ T cells) or MHC class II (for CD4+ T cells) [103].

In the case of colorectal cancer, cDCs can recognize a range of tumor antigens, including neoantigens, which are generated from somatic mutations in cancer cells. These neoantigens are particularly important because they are unique to the tumor and can stimulate a highly specific immune response [104]. cDCs can recognize known cancer antigens such as cancer/testis antigens [105], differentiation antigens [106], mutated antigens or neo-antigens [107,108], and overexpressed oncogenic tumor–self antigens [109]. Under normal circumstances, the immune system has mechanisms in place to prevent autoimmunity, leading to a state of immune tolerance [110]. However, tumors such as CRC can exploit these mechanisms to evade immune detection. Tumors can suppress immune activation through various mechanisms, such as the recruitment of regulatory T cells (Tregs), the expression of immune checkpoint molecules (e.g., PD-L1), and the secretion of immunosuppressive cytokines [110,111]. Classical dendritic cells can overcome these immune evasion strategies by presenting tumor antigens and activating cytotoxic T cells that specifically target tumor cells. Additionally, cDCs can influence the balance of immune tolerance and immunity in the tumor microenvironment, which is essential for generating an effective anti-tumor response [95,112]. cDCs, due to their normal distribution in peripheral blood, the lymphatic system, and tissues, have a superior ability in capturing tumor-associated antigens, cross-presenting exogenous antigens on MHC-I, migrate normally to lymph nodes to start an interaction with naïve T cells, and cross-present antigens to activate CD8+ T cells. These unique features of cDCs enable them to restore immune balance and break through tolerance in the TME [113,114,115,116].

### 4.3. Classical Dendritic Cells in CRC Immunotherapy

The therapeutic use of classical (also known as conventional or myeloid) dendritic cells in CRC immunotherapy can be approached through various strategies, including ex vivo manipulation, DC-based vaccines, and the modulation of the tumor microenvironment to enhance DC function.

#### 4.3.1. Ex Vivo Expansion and Activation of Autologous Dendritic Cells

Dendritic antigens can be isolated to enhance their immune-stimulating abilities. After this activation, the cDCs are reinfused into the patient, where they can present the tumor antigens to T cells and initiate a potent anti-tumor immune response. This approach offers personalization by using autologous cDCs [117,118]. The therapy is tailored to the specific tumor antigens of the individual patient, potentially improving efficacy and reducing the risk of adverse reactions. The ex vivo manipulation allows for the activation of dendritic cells in a controlled environment, optimizing their ability to present tumor antigens and activate a strong immune response [119,120]. By loading cDCs with a broad spectrum of tumor antigens, it is possible to overcome the issue of tumor heterogeneity and enhance immune recognition of diverse tumor cell populations [121].

#### 4.3.2. DC-Based Vaccines

Dendritic cells can be loaded with tumor antigens (e.g., tumor lysates, peptides, or RNA) and administered to the patient to stimulate both CD4+ and CD8+ T cell responses, which are critical for targeting and eliminating tumor cells. This approach offers multiple benefits including targeting multiple tumor antigens, thereby increasing the likelihood of generating an immune response against the tumor, even in the presence of tumor heterogeneity. Effective DC vaccines can induce long-term immunity by stimulating the production of memory T cells, with minimal side effects compared to traditional therapies [122,123].

#### 4.3.3. Modulation of the Tumor Microenvironment

In colorectal cancer, the TME is highly immunosuppressive and presents a significant challenge to the effectiveness of immunotherapy. Tumors often recruit suppressive immune cells such as regulatory T cells (Tregs), myeloid-derived suppressor cells (MDSCs), and tumor-associated macrophages (TAMs), all of which can inhibit the function of DCs and other immune cells [54,124]. Combination strategies to enhance the function of cDCs in CRC immunotherapy involve modulating the TME to promote a more immunogenic environment. The use of immune checkpoint inhibitors, such as anti-PD-1/PD-L1 or anti-CTLA-4 antibodies, can overcome the immunosuppressive effects of the TME and enhance the ability of cDCs to activate T cells [125,126].

These inhibitors have been shown to increase T cell migration and infiltration into tumors and improve responses to DC-based therapies. Cytokines such as GM-CSF (granulocyte–macrophage colony-stimulating factor) can be used to stimulate the maturation and activation of DCs, enhancing their ability to present antigens and stimulate anti-tumor immunity [127]. GM-CSF binds to dendritic cell surface receptors, initiating intracellular signaling (JAK/STAT5, MAPK, NF-κB) that triggers dendritic cell maturation and activation. As a result, increases in the expression of MHC molecules, co-stimulatory molecules (CD80, CD86), and pro-inflammatory cytokines occur, thereby boosting antigen presentation and T cell activation [128,129,130,131].

Targeting Tregs, MDSCs, and TAMs within the TME can help reprogram the immune landscape to promote anti-tumor immunity. For instance, small molecules or antibodies that block the recruitment of suppressive immune cells could increase the effectiveness of DC-based therapies [132,133,134]. When combining DCs and chemotherapy, chemotherapeutic agents can enhance the effectiveness of DCs by inducing tumor cell death and releasing tumor antigens, which are then captured by DCs. Moreover, chemotherapy may help break down the immunosuppressive barriers in the TME, making it more receptive to DC-based therapies [135,136]. In clinical trial settings, radiofrequency thermal ablation of liver cancer showed the transient activation of classical dendritic cells only, not plasmacytoid dendritic cells, which are associated with the anti-tumor proinflammatory cytokines TNF-α and IL-1β [137].

Human CRC liver metastases and microsatellite-stable (MSS) primary CRC have a paucity of T cells and dendritic cells [138]. Combining cryo- or radiofrequency ablation to destroy tumor cells, alongside immune checkpoint inhibitors and dendritic cell loading in preclinical models, resulted in the restoration of the immune balance in the TME, a protective effect against tumor regrowth, and increased tumor-specific T cell responses. The cold from the cryoablation, the heat from the radiofrequency, the precise targeting, and the immune system’s response were all tangible parts of the process [139]. The innovative use of focused ultrasound waves targeted at tumors appears to activate a body-wide immune response directed at cancer-specific molecules, suggesting a novel treatment strategy. Histotripsy, a type of focused ultrasound waves that does not produce heat, can release tumor antigens while maintaining their ability to stimulate the immune system, leading to an abscopal immune response. This technology offers promising advancement in therapies for metastatic and multifocal solid cancers [140,141,142].

#### 4.3.4. Benefits of Classical Dendritic Cells in Colorectal Cancer Immunotherapy

Compared to commonly used monocyte-derived dendritic cells (mo-DCs) in cancer immunotherapy, classical dendritic cells generally offer unique advantages due to their superior ability to efficiently capture and present antigens to T cells in steady-state conditions, particularly in lymphoid organs. This advantage improves the cDCs’ ability to trigger initial immune responses to pathogens and tumors, unlike mo-DCs, which are more involved in inflammation and may be weaker antigen presenters in some cases [143]. In CRC patients, cDCs were discovered to have CD85k expression, a marker associated with immune suppression and tolerance, indicating the potential immunosuppressive impact of the tumor on the host immune microenvironment [144]. Therefore, overcoming the tolerogenicity of cDCs may restore the anti-tumor immune reaction. For these reasons, the use of cDCs in CRC immunotherapy offers a better chance at inducing an anti-tumor immune response, overcoming tumor immune evasion, and targeting tumor heterogeneity, with minimal toxicity [123].

Colorectal cancer, as a heterogeneous disease, may express different antigens depending on the mutations present in individual cancer cells. Variations in colorectal cancer’s genetic makeup, especially in genes such as KRAS, APC, PI3KCA, and BRAF, result in the formation of unique neo-antigens, influencing the immune response against the tumor. The immunogenic, mutated colorectal cancer subtypes exhibit substantial disruptions to immune-modulatory pathways and antigen presentation mechanisms. This involves the biallelic loss of the B2M and HLA genes, caused by copy-number alterations and copy-neutral loss of heterozygosity [12,145,146]. As an example, when a KRAS mutation exists in a CRC patient, it is usually accompanied by more aggressive CRC and a worse overall survival and disease-free survival, compared to those without the mutation. KRAS mutations are associated with a higher likelihood of early relapse after surgical resection or adjuvant therapy [147,148]. cDCs can be loaded with multiple tumor antigens, which allows for the targeting of a broad range of tumor cells, as well as overcoming the immune evasion strategies by activating cytotoxic T cells and promoting an immune response that bypasses tumor-induced suppression. By stimulating memory T cells, DC-based therapies can provide durable anti-tumor immunity, reducing the risk of tumor recurrence and providing patients with long-lasting protection against further disease progression [89,149].

## 5. Natural Killer T (NKT) Cells in CRC Immunotherapy

### 5.1. NKT Cells: Biology and Function

Natural killer T (NKT) cells are a unique subset of T lymphocytes that possess characteristics of both conventional T cells and natural killer (NK) cells. They have properties that are distinct from traditional αβ T cells in that they recognize lipid antigens rather than peptide antigens. NKT cells are divided into two main subsets: type I NKT cells (iNKT cells) and type II NKT cells [96].

**Figure 3:**
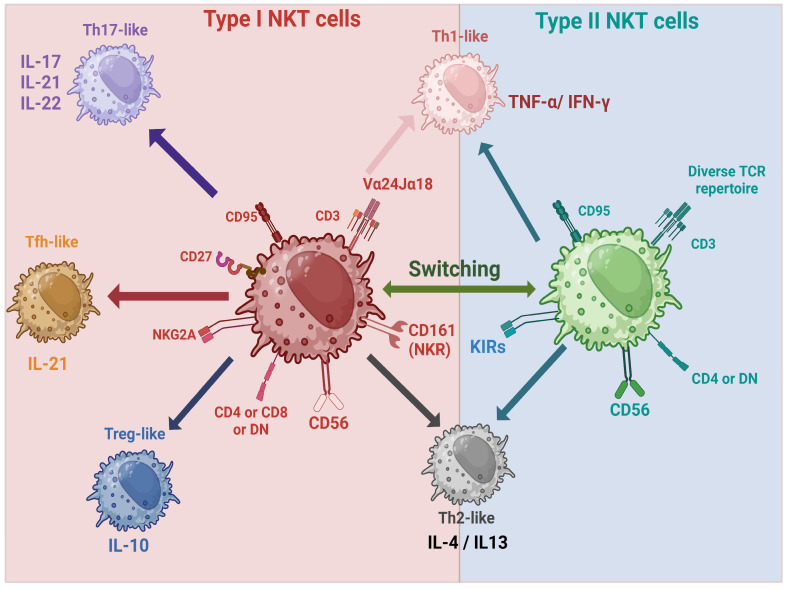
NKT subsets and their secreted cytokines.

Type I NKT cells, also known as invariant NKT (iNKT) cells, express a semi-invariant T cell receptor (TCR) consisting of an α chain (Vα24-Jα18 in humans and Vα14-Jα18 in mice) paired with a β chain (Vβ11 in humans and Vβ8.2 in mice) [150]. These cells recognize lipid antigens presented by the CD1d molecule expressed by antigen-presenting cells, a non-polymorphic MHC class I-like molecule. The antigens that iNKT cells respond to are often glycolipids derived from self or foreign sources, including microbial or tumor-derived antigens. Later, iNKT cells were sub-divided into five subsets with distinction characteristics and functions [151,152] (Figure 3).

Type II NKT cells, on the other hand, are more diverse in their TCR repertoire and recognize a broader range of lipid antigens. These cells tend to have less pronounced effector functions and are not as well understood as iNKT cells. However, both subsets play important roles in immune regulation and responses to pathogens and tumors. Currently, Type II NKT are subdivided into two subsets with unknown functions and distributions [153,154].

### 5.2. Activation and Cytokine Production

Upon recognition of lipid antigens presented by CD1d, iNKT cells undergo rapid activation and produce a wide array of cytokines, including IFN-γ, IL-4, TNF-α, and IL-17. The ability of iNKT cells to produce both Th1 (e.g., IFN-γ) and Th2 (e.g., IL-4) cytokines allows them to modulate both innate and adaptive immune responses. The cytokines they produce can help recruit DCs, macrophages, and conventional T cells, thereby amplifying the immune response [155]. The ability of iNKT cells to produce IFN-γ and IL-4 upon activation enables them to influence the immune microenvironment, facilitating anti-tumor immunity while simultaneously modulating immune tolerance. This makes iNKT cells especially valuable in cancer immunotherapy, where immune regulation is crucial for both enhancing anti-tumor responses and preventing autoimmunity [156,157].

### 5.3. Anti-Tumor Activity of NKT Cells

NKT cells, particularly iNKT cells, exhibit direct anti-tumor activity through several mechanisms. Their rapid activation and production of cytokines, such as IFN-γ and TNF-α, can trigger the killing of tumor cells, either directly or indirectly. iNKT cells can activate other cytotoxic immune cells, such as NK cells and CD8+ T cells, which then target and destroy cancer cells. Additionally, iNKT cells can enhance the effectiveness of DCs in presenting tumor antigens to conventional T cells, thereby strengthening the adaptive immune response [158].

One of the most notable features of iNKT cells is their ability to recognize and respond to glycolipid antigens that are often overexpressed or uniquely expressed in tumor cells [159]. These glycolipids, such as α-galactosylceramide (α-GalCer), a synthetic ligand for iNKT cells, have been shown to activate NKT cells and induce strong anti-tumor responses. This provides a mechanism through which NKT cells can directly target tumors that overexpress specific glycolipids, a feature that is often found in a variety of cancers, including CRC [160,161]. The potential for NKT cells to be used in immunotherapy lies in their similarities to T cells, including their proximity to cancer cells and high levels of granzyme B expression, like T cells [162].

### 5.4. NKT Cells in CRC Immunotherapy

Given their ability to produce pro-inflammatory cytokines and activate other immune cells, NKT cells present a promising strategy for CRC immunotherapy. Unlike conventional T cells, which require antigen presentation via MHC molecules, NKT cells recognize lipid antigens presented by CD1d molecules [97,163]. Numerous tumors, including CRC, exhibit alterations in lipid metabolism, leading to the expression of tumor-specific glycolipids that can serve as targets for NKT cell recognition [164]. Tumor cell proliferation is accelerated by increased lipid synthesis and uptake, which provide the necessary building blocks for membranes and energy production, and thus driving tumor growth and development [165,166]. The alteration in lipid metabolism leads to a change in the glycolipids expressed on the surface of tumor cells that can act as tumor-associated antigens. The aberrant glycosylation patterns in CRC can lead to the formation of novel lipid antigens, making these molecules distinct and recognizable by NKT cells. These tumor-specific glycolipids in CRC bind to CD1d and are presented to NKT cells, which then produce cytokines like IFN-γ, enhancing cytotoxic T cell activity, and recruiting other immune cells to the tumor microenvironment [167,168,169,170].

The potential benefits of utilizing NKT cells in CRC immunotherapy stem from their ability to directly target tumor cells and modulate the immune microenvironment [99]. In addition to their cytotoxic activity, NKT cells can activate NK cells, CD8+ T cells, and DCs, thereby amplifying the immune response and enhancing tumor cell destruction (Table 1). Their ability to regulate the immune response and produce both Th1 and Th2 cytokines positions them as key players in modulating the TME and overcoming tumor-induced immune suppression [97,171].

#### 5.4.1. Adoptive Transfer of NKT Cells

One of the most promising strategies for harnessing the anti-tumor potential of NKT cells in CRC is through adoptive transfer. In this approach, NKT cells are isolated from the patient or a healthy donor, expanded ex vivo, and then reinfused into the patient to promote anti-tumor immunity. This adoptive cell therapy (ACT) has been successfully used in other cancers, such as leukemia and melanoma, and shows potential for CRC [177,180].

#### 5.4.2. NKT Cell-Based Vaccines

The anti-tumor potential of NKT cells can be harnessed through the development of NKT cell-based vaccines. These vaccines aim to stimulate the patient’s own NKT cells to recognize and respond to tumor-associated glycolipids [181]. The use of α-GalCer, a potent NKT cell agonist, as a vaccine component has been shown to induce strong NKT cell activation and enhance anti-tumor immunity in preclinical models of CRC [182]. In addition to α-GalCer, other glycolipids that are expressed on CRC cells or in the TME could be used as targets for NKT cell-based vaccines [181,183]. These vaccines could be combined with adjuvants to enhance NKT cell activation and promote long-term immunity. Combining NKT cell-based vaccines with other immunotherapies, or locoregional therapies in addition to chemotherapies, could provide synergistic effects and improve treatment outcomes [169,182]. All the clinical trials that used NKT cells for immunotherapy are listed in Table 1.

### 5.5. Challenges and Limitations in the Clinical Use of NKT Cells for CRC

The tumor microenvironment (TME) of CRC is typically immunosuppressive and creates a hostile environment for immune cells, including NKT cells, limiting their ability to effectively target and eliminate tumor cells [44,184]. Strategies to overcome these immunosuppressive mechanisms are critical for improving the efficacy of NKT cell-based therapies. Combining NKT cell therapies with immune checkpoint inhibitors, which block the inhibitory signals on T cells and NKT cells, has shown promise in enhancing anti-tumor immunity in CRC. Additionally, targeting the TME with agents that deplete or reprogram suppressive immune cells may improve the ability of NKT cells to function effectively in the tumor [185,186]. CRC is a heterogeneous disease with a wide range of genetic and epigenetic alterations. The epigenetic alterations include hypermethylation of tumor suppressor genes or hypomethylation of oncogenes, loss of histone acetylation or aberrant histone methylation, dysregulation of non-coding RNAs (miRNA and lncRNA), and chromatin remodeling [187,188,189]. This heterogeneity poses a significant challenge for NKT cell therapies, as the expression of tumor-specific glycolipids may vary between patients or even within different areas of the same tumor [190,191]. To overcome this limitation, personalized approaches that identify tumor-specific glycolipids and tailor NKT cell therapies accordingly are needed. Moreover, targeting a broad range of glycolipids expressed across different CRC subtypes may improve the effectiveness of NKT cell-based therapies. Combining NKT cells with other immune-based therapies may also enhance their ability to target a broader range of tumor antigens and improve clinical outcomes [177,192]. The ex vivo expansion of NKT cells remains a challenge, as these cells are relatively rare in the peripheral blood. Efficient methods for isolating, expanding, and activating NKT cells are crucial for developing clinically viable therapies [193]. Moreover, once infused into the patient, NKT cells may have limited persistence, which can reduce the long-term benefits of adoptive transfer therapies. Developing strategies to enhance NKT cell survival and expansion in vivo, as well as improving their homing to tumor sites, is an important area of ongoing research [177,192,193].

## 6. cDCs and NKT Cell Combination Therapy: Unique Benefits and Promising Outcomes for CRC Therapy

The combination of classical or conventional dendritic cells and invariant natural killer T (iNKT) cells in solid cancer therapy has shown promise in overcoming the immune-suppressive tumor microenvironment by augmenting tumor antigen presentation and stimulating a robust T cell-mediated immune attack [194]. The use of autologous cDCs loaded with tumor antigens, combined with iNKT cells, was shown to lead to enhanced immune activation, increased infiltration of cytotoxic T cells into tumors, and favorable outcomes in in vitro and in vivo studies [157,169,195]. The reciprocal relationship between dendritic cells and NKT cells is a subject of intense study, especially within the context of infectious diseases. Activation signals from DCs are received by NKT cells, which frequently give feedback to the DCs. In addition to affecting DCs directly, NKT cells can also impact DC function by interacting with innate immune cells like NK cells [196].

A phase I clinical trial was conducted in advanced pancreatic cancer patients who failed to respond to the first line of chemotherapy. Patients treated with a fusion of iNKT cells with PD-1+ CD8 T cells had a reduced tumor burden and prolonged survival time [197].

**Figure 4:**
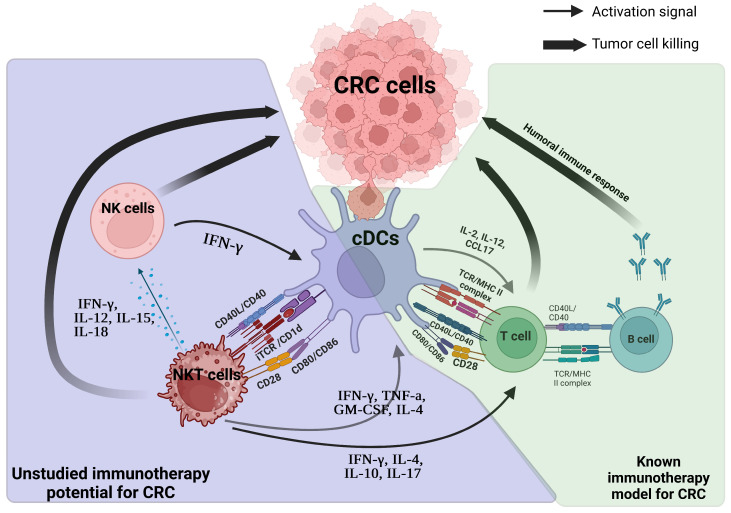
Crosstalk between cDCs and NKT cells as an alternative pathway for immune activation and cancer eradication. Black bold arrows indicate cytotoxicity towards cancer cells.

Moreover, this combination therapy might offer a more targeted immune response, lowering the risks of the off-target effects and systemic toxicity commonly associated with traditional cancer treatments like chemotherapy and radiation [198]. These findings underscore the promise of cDCs and iNKT cell-based immunotherapies as a novel strategy for treating solid tumors. The combination of cDCs and NKT has not been clinically studied, although hypothetically, the crosstalk between these two important cells could yield favorable synergistic responses. This combination may be a new promising approach to overcome cancer resistance to therapies and metastatic solid cancers (Figure 4).

## 7. Conclusions

Several challenges in CRC immunotherapy remain, including the tumor heterogeneity, immunosuppressive TME, and low mutational burden of CRC. Cellular immunotherapy using classical dendritic cells and NKT cells are potential options for improving responses by increasing the recognition of tumor antigens and priming cytotoxic immune responses independent from the conventional T cell pathway in CRC. NKT cells may be a unique immune reaction orchestrator that is resistant to tumor immune suppression and is suitable use in novel cellular immunotherapies for cancers when combined with classical dendritic cells. Studies have shown the potential of cDCs and NKT cell-based therapies alone in CRC, but not in combination. The addition of locoregional interventions, targeting of the tumor microenvironment, and combinations of these therapies may further increase this response by increasing antigen recognition and processing after cell death.

## Figures and Tables

**Table 1:** Clinical trials using NKT cells in interventional cancer immunotherapies.

Intervention	Conditions	Study Status	Phases	NCT No.	Date	Results	Ref.
Synthetically derived agonist of iNKT cells (IMM60), Pembrolizumab	NSCLC, Melanoma	TERMINATED	Phase I/II	NCT05709821	2023/11/15 to 2024/04/22	In 8 melanoma patients: 2 lesions were completely resolved, 1 lesion showed 69% decrease in size, 10 lesions were stable, and 6 lesions showed >20% increase in size. Cytokine analysis showed iNKT and NK activation and increases in dendritic and CD86+ B cells.	[172]
Estimation of percentage of Treg, Th17, NKT cells in serum and tumor tissue	Ovarian Cancer, Unexplained Infertility	COMPLETED	Observational	NCT03779399	2011/12/01 to 2016/12/31	In benign ovarian tumors: increased number of iNKT cells detected. In ovarian cancer patients: higher number of iNKT cells in tumor tissue and negative correlation between the CA125 serum marker and NKT cells.	[173]
Infusion of iNKT cells and CD8+T cells	Advanced Solid Tumor	COMPLETED	Phase I/II	NCT03093688	2017/03/01 to 2023/06/30	Extended overall survival time to over 12 months in 6 of the 9 patients. Elevated number of CD8^+^ T cells after the first course.	[174]
iNKT cells, IL-2, Tegafur	Hepatocellular Carcinoma	COMPLETED	Phase I	NCT03175679	2017/04/01 to 2019/03/30	Production of greater quantities of T-helper 1 (Th1) cytokines. Increase in number of circulating iNKT cells and activated NK cells after iNKT cell infusion. Side effects were resolved without treatment. Four patients were progression-free at 5.5, 6, 7, and 11 months after therapy, and one patient was alive and without tumor recurrence at the last follow-up.	[175]
iNKT cells, recombinant Il-2, TAE/TACE	Hepatocellular Carcinoma	COMPLETED	Phase II	NCT04011033	2018/03/01 to 2023/10/01	Median PFS, ORR, DCR, and mean lymphocyte count were significantly higher in TAE-iNKT patients compared with TAE patients.	[176]
Allogenic NKT: agenT-797	Relapsed/Refractory/ Multiple Myeloma	COMPLETED	Phase I	NCT04754100	2021/03/29 to 2023/05/31	No side effects were recorded up to a dose of 1 × 10^9^ cells.	[177]
Allogenic NKT: agenT-797, ICI	Relapsed/Refractory Solid Tumors	COMPLETED	Phase I	NCT05108623	2022/01/28 to 2024/01/02	Unconventional T cells became activated in response to cellular or inflammatory stimuli rather than tumor recognition.	[178]
Reference values for circulating natural killer T-like (NKT) cells	Healthy	COMPLETED	NA	NCT06450743	2024/05/03 to 2024/05/31	No results posted.	-
Estimation of number of NKT cells in gut biopsies and blood samples	Inflammatory Bowel Disease, Primary Sclerosing Cholangitis	COMPLETED	NA	NCT02884557	2013/05 to 2019/08/28	No results posted.	-
Infusion of iNKT cells	Malignant Solid Tumor	COMPLETED	Phase I/II	NCT03551795	2018/01/01 to 2022/03/31	No results posted.	-
INKT, GM-CSF	Malignant Melanoma	COMPLETED	Phase I	NCT00631072	2008/02 to 2015/04	No results posted.	-
GINAKIT cells + Etanercept	Neuroblastoma	RECRUITING	Phase I	NCT03294954	2018/01/18 to 2040/08/10	CAR-NKT cells expanded in vivo and localized to tumors and, in one patient, induced regression of bone metastatic lesions (PMID: 33046868). The objective response rate was 25% of patients: 16% showed partial responses and 8% showed a complete response CD62L^+^NKT cells correlated with CAR-NKT cell expansion in patients and was higher in responders than non-responders.	[179]
CD19.CAR-aNKT cells	Refractory B Cell Non-Hodgkin Lymphoma, Relapsed ALL/CLL|NHL	RECRUITING	Phase I	NCT03774654	2020/06/22 to 2035/03/01	No results posted.	-
CAR-NKT cell treatment	Advanced Malignant Solid Tumors	RECRUITING	Phase I	NCT06728189	2024/11/14 to 2026/10/17	No results posted.	-
Cyclophosphamide + Fludarabine + Infusion of CAR-NKT cells	Solid Tumors	RECRUITING	Phase I	NCT06394622	2024/04/11 to 2026/06/28	No results posted.	-
Cyclophosphamide + Fludarabine + Infusion of CAR-NKT cells	Renal Cell Carcinoma	RECRUITING	Phase I	NCT06182735	2023/07/17 to 2025/01/28	No results posted.	-
Allogeneic NKT cells expressing CD19-specific CAR	B-Cell Malignancies	RECRUITING	Phase I	NCT05487651	2022/10/01 to 2024/12	No results posted.	-
iNKT cells, PD-1, Regorafenib	Hepatocellular Carcinoma	RECRUITING	Phase II	NCT05962450	2023/10/26 to 2025/08/01	No results posted.	-
Allogenic NKT cells: agenT-797, Botensilimab, Balstilimab, Ramucirumab, Paclitaxel	Esophageal, Gastric, or Gastro-esophageal Junction Cancer	RECRUITING	Phase II	NCT06251973	2024/02/01 to 2027/08/01	No results posted.	-
NKT cells	Melanoma	UNKNOWN	Phase I	NCT02619058	2015/10 to 2017/10	No results posted.	-
NKT cells	Breast Cancer, Glioma, HCC, SCLC, Pancreatic Cancer, CRC, Prostate Cancer	UNKNOWN	NA	NCT01801852	2013/01 to 2017/06	No results posted.	-
Natural killer T cells	NSLC, GC, HCC, CRC	UNKNOWN	Phase I/II	NCT02562963	2015/11 to 2024/12	No results posted.	-
Infusion of natural killer and natural killer T cells	Non-small Cell Lung Cancer	UNKNOWN	Phase I	NCT03198923	2017/09/13 to 2020/12	No results posted.	-
Expansion of invariant NKT cells as a cell immunotherapy	Allogeneic Hematopoietic Stem Cell (HSC) Transplantation	UNKNOWN	Observational	NCT03605953	2018/10/01 to 2021/04/01	No results posted.	-
hCD19.IL15.CAR-iNKT	ALL, CLL, B cell Lymphoma	UNKNOWN	Phase I	NCT04814004	2021/03/19 to 2024/04/01	No results posted.	-

## Data Availability

Not applicable.
